# Off-Site Indoor Localization Competitions Based on Measured Data in a Warehouse †

**DOI:** 10.3390/s19040763

**Published:** 2019-02-13

**Authors:** Ryosuke Ichikari, Katsuhiko Kaji, Ryo Shimomura, Masakatsu Kourogi, Takashi Okuma, Takeshi Kurata

**Affiliations:** 1 National Institute of Advanced Industrial Science and Technology (AIST), Tsukuba 305-8560, Japan; r.ichikari@aist.go.jp (R.I.); r.shimomura@aist.go.jp (R.S.); m.kourogi@aist.go.jp (M.K.); takashi-okuma@aist.go.jp (T.O.); 2 Faculty of Information Science, Aichi Institute of Technology, Toyota 470-0392, Japan; kaji@aitech.ac.jp; 3 Graduate Schools of Systems and Information Engineering, University of Tsukuba, Tsukuba 305-8573, Japan; 4 Sumitomo Electric Industries, Ltd., Osaka 541-0041, Japan

**Keywords:** pedestrian dead-reckoning (PDR), vehicle dead-reckoning (VDR), xDR, warehouse, indoor localization competition, PDR Challenge, xDR Challenge, IPIN, forklifts

## Abstract

The performance of indoor localization methods is highly dependent on the situations in which they are used. Various competitions on indoor localization have been held for fairly comparing the existing indoor localization methods in shared and controlled testing environments. However, it is difficult to evaluate the practical performance in industrial scenarios through the existing competitions. This paper introduces two indoor localization competitions, which are named the “PDR Challenge in Warehouse Picking 2017” and “xDR Challenge for Warehouse Operations 2018” for tracking workers and vehicles in a warehouse scenario. For the PDR Challenge in Warehouse Picking 2017, we conducted a unique competition based on the data measured during the actual picking operation in an actual warehouse. We term the dead-reckoning of a vehicle as vehicle dead-reckoning (VDR), and the term “xDR” is derived from pedestrian dead-reckoning (PDR) plus VDR. As a sequel competition of the PDR Challenge in Warehouse Picking 2017, the xDR Challenge for Warehouse Operations 2018 was conducted as the world’s first competition that deals with tracking forklifts by VDR with smartphones. In the paper, first, we briefly summarize the existing competitions, and clarify the characteristics of our competitions by comparing them with other competitions. Our competitions have the unique capability of evaluating the practical performance in a warehouse by using the actual measured data as the test data and applying multi-faceted evaluation metrics. As a result, we successfully organize the competitions due to the many participants from many countries. As a conclusion of the paper, we summarize the findings of the competitions.

## 1. Introduction

One of the most important keys for making services based on the Internet of Things (IoT) or Internet of Humans (IoH) succeed is an accurate and efficient localization of things and humans. In an indoor environment, satellite signals for the global navigation satellite system cannot be reached. Therefore, infrastructures for indoor localization should be individually installed for each site. Through the active discussions of researchers in academic venues, such as in international conferences on indoor localization and indoor navigation (IPIN) [1] and ACM International Joint Conference on Pervasive and Ubiquitous Computing (UbiComp) [2], many methods and systems have been developed for indoor localization. However, service developers need to carefully choose a suitable indoor localization method from the several available options. The performance of indoor localization methods is highly dependent on the situation in which they are used. Generally, it is difficult to find an optimal solution from only the information obtained from the localization system developers. To fairly evaluate the existing localization methods, tracking competitions have been held by preparing shared data or controlled environments [3,4,5,6,7,8,9].

Pedestrian dead-reckoning (PDR) is a relative tracking method that is mainly used for indoor localization. We consider PDR to be a dominant method of indoor localization, because it can individually track the target position without relying on other infrastructures. However, comparing PDR methods is very difficult due to the nature of relative tracking. The performance of an integrated location system based on PDR depends on many aspects such as the accuracy of the relative tracking by PDR, the accuracy of the initial position and direction, and the accuracy of the error correction methods for reducing the accumulated error. To discuss and standardize PDR benchmarking, a PDR benchmark standardization committee has been established [10], which hosted the competitions that are introduced in this study. 

In this paper, we introduce our indoor localization competitions, which are named the PDR Challenge in Warehouse picking (hereinafter the PDR Challenge 2017) and the xDR Challenge for Warehouse Operations (hereinafter the xDR Challenge 2018), for tracking workers and vehicles in a warehouse scenario. Before introducing the details of the competitions, in Section 2, we briefly summarize the existing competitions and clarify the characteristics and focuses of our competitions. In Section 3, we introduce the PDR challenge 2017, which was conducted as an official competition track of IPIN2017, and its results. In Section 4, we introduce the xDR Challenge 2018, which was conducted as a sequel competition to the PDR Challenge 2017, by reflecting on the findings from the PDR Challenge 2017. In Section 5, we introduce the disclosure of the evaluation program. Finally, in Section 6, we conclude the paper and introduce plans for future competitions.

## 2. Survey of the Existing Indoor Localization Competitions and Comparison with PDR Challenges

### 2.1. Overview of the Existing Indoor Localization Competitions

As described in Section 1, many indoor localization competitions have been held for various purposes under various scenarios. In this section, we survey the existing competitions and summarize them in Table 1. This survey aims to clarify the characteristics of our PDR Challenge 2017 and xDR Challenge 2018.

As the existing on-site and off-site indoor localization competitions, we select the following three competitions series as popular and dominant indoor localization competitions.

- PerLoc Competition, which was organized by National Institute of Standards and Technology (NIST) [3,4];
- EvAAL (Evaluating AAL Systems through Competitive Benchmarking)/IPIN Competitions [5,6,7];
- Indoor Localization Competitions, organized by Microsoft [8].

We compare the above external competitions with the following series of competitions regarded as PDR Challenges, which were organized by the PDR benchmark standardization committee.

- PDR Challenge (original, held at UbiComp2015/International Symposium on Wearable Computers (ISWC2015) [9]);
- PDR Challenge in Warehouse Picking 2017 (PDR Challenge 2017);
- xDR Challenge for Warehouse Operations 2018 (xDR Challenge 2018).

### 2.2. Survey Items

To organize the survey of the competitions, we list the following items. In Section 2.3, the competitions are surveyed according to the survey items, and the survey items are introduced in detail.

#### 2.2.1. Scenarios

To focus on the target of the competitions, the organizers of the competition tend to assume a specific scenario and target of the competition, and prepare the test environment based on that scenario. Although the scenario of the competition is not always explicitly stated, we introduce the assumed scenarios for each competition.

#### 2.2.2. Types of Contained Motions

This survey item describes the possible motion types enacted by the testers of the competition. If various motions are contained in the dataset for the off-site competition or are assumed to be enacted by the testers for the on-site competition, the contestants’ localization methods are required to be capable of handling various motions or detecting changes in the motion types. Therefore, this survey item checks the ability of the competition for evaluating the robustness and flexibility for various motions.

#### 2.2.3. Testers

The tester for the competition is another important factor concerning the condition of the competition and quality of the test data. Some competitions keep consistent testers for maintaining the same condition. However, it is difficult to perfectly unify the condition of the competition. At some competitions, participants themselves can be the testers. For an off-site competition, participants are assumed to compete with common test data. Therefore, there is no problem caused by the inconsistency of the testers. In most cases, testers are assigned within the group of organizers. In some other competitions, external testers are assigned for guaranteeing equitability and generality.

#### 2.2.4. Competition Types

There are two types of competitions in the research community on indoor localization. One type is an on-site competition, where the organizers prepare controlled environments for testing, which are measured carefully in advance. The competitors run their algorithms or systems in the testing environment. The other type is an off-site competition, wherein instead of physically testing in the test environment, the organizers prepare test data, which are shared with the competitors. The competitors apply their algorithms or methods to the test data and submit the results to the organizers.

#### 2.2.5. Target Types of Methods for the Competition

The target types of methods can be limited by the regulation of the competitions, configuration of the test environment, and data provided by the organizers. For example, localization methods using Wi-Fi signals from Wi-Fi access points can be applied only if Wi-Fi signal data are available for the contestants. This survey item checks the types of methods that are assumed as the target methods.

#### 2.2.6. Amount of Test Data

This survey item is for off-site competitions and checks the amount of test data provided for the participants. This amount determines the number of trajectories that are required to be submitted. If the number of trajectories is large, a variety of data are expected to be included in the test data, and over-tuning for a specific test data can be avoided.

#### 2.2.7. Time Length of the Test (Data)

This checks length of time of the test data or an actual on-site test, and is related to the difficulty of the competition. If the length of the test data increases, it is expected that a great variety of data is included and the total trajectory optimization becomes harder. Methods that are categorized as relative tracking methods often suffer from error accumulation. Therefore, the length of the test data is especially crucial for them.

#### 2.2.8. Space of the Test Environment or Length of the Test Course

Similar to the time length of the actual test or test data, the space of the test environment and the length of the test courses are also related to the difficulty of the competition. If the test space is large or the test course is long, relative tracking methods are required to handle the problem of error accumulation. The availability of absolute localization often becomes a problem because the arrangement of infrastructure for covering a wide area becomes difficult.

#### 2.2.9. Evaluation Methods

This survey item checks how to evaluate the submitted trajectory or recorded result obtained on-site. The evaluation is based on a comparison of positions between the ground truth. This also checks how to quantify the results evaluated at the multiple evaluation points by using statistical measures.

#### 2.2.10. History of Competitions/Remarks about the Competitions

Some competitions introduced here have a long history. Holding competitions continuously is also important for gathering many participants and spreading evaluation frameworks. This survey item introduces the competition’s history. In the case of PDR Challenges, we have held three competitions in total, for which notable remarks are introduced instead.

#### 2.2.11. Number of Participants

The number of participants is related to the degree of recognition and difficulty for winning the competitions. If the number of participants is large, the competition is widely recognized, and the difficulty for winning the competition is hard. A competition with a long history tends to gather many participants. If the competitions are too challenging, the potential competitors might hesitate in joining them. 

#### 2.2.12. Award and Prize for First Place

In most competitions, the winner is awarded. Winners can receive an award and/or a prize. This factor is related to the incentive for joining the competition. A competition with rich awards is expected to attract many participants.

#### 2.2.13. Connection with Academic Conference

Many competitions have been held in conjunction with academic conferences. If a competition can be successfully held in conjunction with a prestigious academic conference, the competition can also obtain a prestigious position with the endorsement of the academic conference. This also helps with the announcement of the event for the research community in the academic conference. Some conferences encourage submitting related papers with the system that is used for the competition. Therefore, the participants can utilize the opportunity for scientific publication in such a case.

### 2.3. Comparison of the Existing Indoor Localization Competitions

#### 2.3.1. PerfLoc

PerfLoc is an indoor localization competition that was held in 2017 by NIST, which is a governmental research organization for standardization in the United States. PerfLoc adopts a two-stage competition. First, an off-site competition is held, and then, top performers are invited to the on-site evaluation through a live demonstration. The main scenario for PerfLoc is an emergency scenario, which is one of the most important concerns for governmental organizations in the United States. The total number of scenarios is about 30, and the dataset for the off-site competition includes various motion types, such as walking, running, backwards/side-step, crawling, pushcart, and using elevators. The test data are measured using four smartphones mounted on the tester’s arm. The included motion types and the arm-mounted measuring style are uniquely reflected by the emergency scenario. The datasets used for the off-line competition are measured in four buildings with a total area of 30,000 m^2^. The evaluation metric for calculating the score is based on the 95th percentile value of the three-dimensional (3D) error (which is defined as SE95 in the International Organization for Standardization (ISO)/the International Electrotechnical Commission (IEC) 18305 standards [11]), which is calculated at the check points, and defined in advance. As the awards, the winner ranking first can get 20,000 United States (US) dollars; however, only US citizens or permanent residents are eligible to be awarded. This rich award is one of the most remarkable characteristics of PerfLoc. A portal website is also prepared for providing the dataset, automatic evaluation, and ranking on the Internet. As a result of these efforts, PerfLoc attracted 169 teams as pre-registrants due to providing the dataset. Finally, 16 teams submitted the result. The competition is not directly related to an academic conference; the evaluation framework is based on the ISO/IEC 18305 standard.

#### 2.3.2. EvAAL/IPIN Competition

EvAAL stands for evaluating Ambient Assisted Living (AAL) systems through competitive benchmarking. The EvAAL competition has a relatively long history. The EvAAL competitions have been conducted since 2011 under the AAL scenario. In 2014, EvAAL collaborated with the Electronics and Telecommunications Research Institute (ETRI) of South Korea for holding a competition during IPIN at Busan, Korea. Since then, IPIN competitions have been adopting EvAAL’s framework. EvAAL/IPIN competitions comprise multiple tracks (mostly four), which are categorized by off-site or on-site tracks and types of devices that are used for the localization. The test course for measuring data for off-site competitions and that for evaluating the systems in on-site competitions are predefined. The measurement process, or on-site evaluations, is operated by the competitors themselves or other testers assigned by the organizers while stepping on check points defined and measured in advance. The contained motions include various types such as walking, taking stairs, taking an elevator, using a phone, and lateral movements. The evaluation metric for calculating the score is based on the 75th percentile value of the 3D error (called SE75 in the ISO/IEC 18305 standard), and calculated at predefined check points. The location and area of the target environments vary from year to year. For example, in 2016, the size of the testing environment was 7000 m^2^ for tracks 1 and 2. The total number of participants in the four tracks was 24. As described, the EvAAL/IPIN competition is held in conjunction with the IPIN conference, and the participants are encouraged to submit papers on the method that they use in the competition. The amount of the cash award for the first place depends on the year; for example, it was 1500 euros in 2016.

#### 2.3.3. Microsoft Indoor Localization Competitions

Microsoft has been conducting indoor localization competitions at the International Conference on Information Processing in Sensor Networks since 2014. This competition comprises only on-site tracks, which are divided into two-dimensional (2D) and 3D tracks. In the 2D track, the participants are required to estimate the 2D position by using any method without relying on infrastructure. On the other hand, in the 3D track, the participants are required to estimate the 3D position of the target by using any method, including the limited number of positional infrastructures. Note that in 2017, the winner of the 3D track achieved an incredibly great score with a 0.03-m average absolute error by using light detection and ranging (LiDAR). As a result, in 2018, the use of LiDAR was prohibited. The number of events, number of participants, and numerical score that the winner achieved are impressive. However, the competition does not seem to be based on realistic scenarios, and the competition area is relatively small; for example, it was 600 m^2^ in 2018. Another remarkable difference from other competitions is that the participants can operate their own system during the on-site test. The amount of the cash award for first place was 1000 US dollars in 2018. 

### 2.4. Competitions in PDR Challenge Series

#### 2.4.1. PDR Challenge@UbiComp/ISWC2015 [9]

PDR Challenge is a competition series that is hosted by the PDR benchmark standardization committee. The following are its characteristics: the main target algorithm for the competition is PDR, and the competitions are held under realistic scenarios. The original PDR Challenge was held at a prestigious international conference, which is named UbiComp/ISWC 2015, under the scenario of pedestrian navigation. This competition was held as an off-site competition, but data measurements were carried out on-site. The participants of UbiComp/ISWC contributed to the data measurement. The participants of the competitions were asked to submit the source code of the methods, and the submitted trajectories were automatically evaluated. The motion type was walking in an indoor environment while watching a smartphone display. The length of the test course was about 100 m each. The data included 229 sequences owing to 90 participants. Therefore, the dataset had various walking motions derived from the differences between the people. The measured data were archived as the Human Activity Sensing Consortium (HASC) corpus [12] and shared with the researchers. The evaluation metric was an average error. The number of final participants was five. The prize for the winner ranking first was 50,000 yen. 

#### 2.4.2. PDR Challenge in Warehouse Picking [13]

The PDR Challenge in Warehouse Picking 2017 was held as an official competition track of IPIN 2017 under the scenario of the behavior measurement of employees who pick items from shelves in a warehouse. The PDR Challenge 2017 was held as an off-site competition. One of the main characteristics of this competition was that the test data used were measured during a real picking operation at an actual warehouse. Therefore, the measured test data included various types of motion during the real picking operation, such as picking items, side-stepping, walking, and pushing a cart. As we regard this competition as similar to that in the PDR Challenge series, the target method for this competition is a PDR-based indoor localization method. To integrate with PDR for cancelling the PDR error, Bluetooth Low Energy (BLE) beacons are arranged in the warehouse, and a log of the warehouse management system (WMS) was provided to the competitors. The WMS is utilized for managing items in the shelves and providing an operation order for the picking workers. When the picking workers pick an item from the shelf, they scan the barcode by using a handy terminal for checking the shelf from which they were asked to pick the item. The WMS log corresponds to the time-series data of the worker positions. In the PDR Challenge 2017, the WMS data are used for the ground truth of the position of the target worker, and provided for the participants of the competition for the partial reference data of the position for error correction. In other words, the target method of the PDR Challenge is integrated indoor localization with PDR, BLE, and a WMS. Another important characteristic of the PDR Challenge 2017 is the adaptation of multi-faceted evaluation metrics for the practice performance of the PDR-based localization method in a warehouse scenario. The size of the target warehouse is about 8000 m^2^. The winner in first place gets 10,000 yen and a prize. The competition is very challenging, because the competitors are required to handle real data. Fortunately, we successfully attracted five participants from four countries. Our previous conference paper [13] introduced a preliminary report on the PDR Challenge 2017 and a preliminary plan for the xDR Challenge 2018. In this paper, we will introduce their final detailed reports.

#### 2.4.3. xDR Challenge for Warehouse Operations [13]

The xDR Challenge for Warehouse Operations 2018 was held as a sequel competition of the PDR Challenge 2017. This year, the competition was not held as an official competition track of IPIN’s competition. We organized a special session on the indoor localization competitions, and the result of the xDR Challenge was announced during this session. The scenario and evaluation frameworks were the same as those in the PDR Challenge 2017. The xDR Challenge 2018 was also held as an off-site competition. In addition to tracking employees who moved in the warehouse on foot, the forklift driven by the employees was also tracked as the tracking target. We call the dead-reckoning of a vehicle ‘vehicle dead-reckoning’ (VDR) [14]. This year’s competition was renamed as the xDR Challenge, which indicates a competition for PDR and VDR. The area of the competition is exactly same as that of the target warehouse of the PDR Challenge 2017. The evaluation metrics are also almost same as those of the PDR Challenge 2017. We obtained some feedback at the PDR Challenge 2017. To reflect this feedback, the weights for the calculation comprehensive evaluation and the evaluation of the PDR error accumulation were also modified. In addition, the provided subsidiary information and reference data were enriched. From the next section, we will introduce the details of the PDR Challenge 2017, as well as the findings from the PDR Challenge 2017 and xDR Challenge 2018, and reflections on these findings.

## 3. PDR Challenge in Warehouse Picking

### 3.1. Overview of the PDR Challenge in Warehouse Picking

The PDR Challenge 2017 was held as an official tracking competition during the IPIN 2017 conference, with the aim of evaluating PDR-based indoor localization methods under a realistic scenario. The PDR Challenge 2017 adhered to the original format of the PDR Challenge that was held at UbiComp/ISWC 2015 under the scenario of indoor navigation. On the contrary, the assumed scenario of the PDR Challenge 2017 was tracking employees during the picking operation in the warehouse. 

The data used for the PDR Challenge 2017 were measured during the actual picking operation in an actual warehouse. Therefore, the measured behaviors were not limited to walking. They included other operations than walking, such as pushing carts, picking objects, driving forklifts, and using handy terminals. The duration of the data measurement was approximately three hours. However, the competitors could grasp the entire data with unveiled correct reference points and apply a global optimization algorithm to them for improving the indoor localization accuracy. 

**Figure 1:**
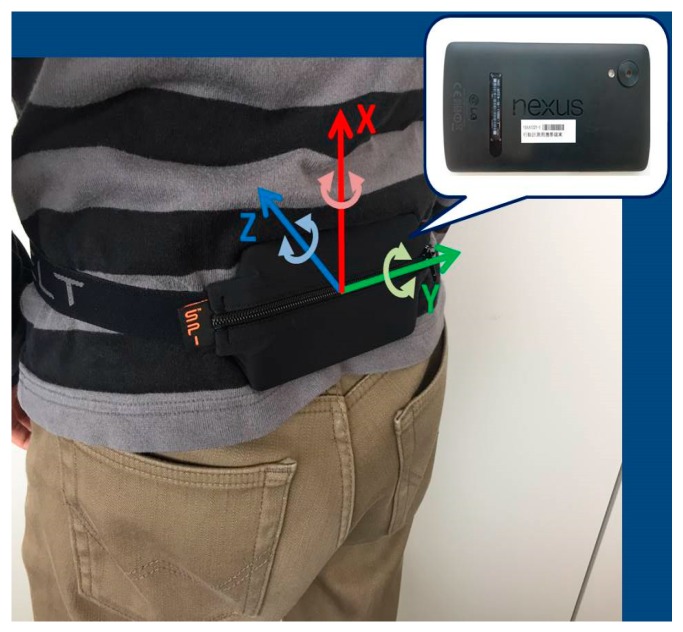
To measure sensor data required for pedestrian dead-reckoning (PDR), we asked the employees to carry the smartphone during the operation.

The sensor data were measured by having employees carry a smartphone in a pouch, as shown in Figure 1. The logging application installed in the smartphones recorded the following data.

- Raw sensor data for PDR: angular velocity, acceleration, magnetism, and atmospheric pressure (at 100 Hz).
- Received signal strength indicator (RSSI) from the BLE beacons (at about 2 Hz).

**Figure 2:**
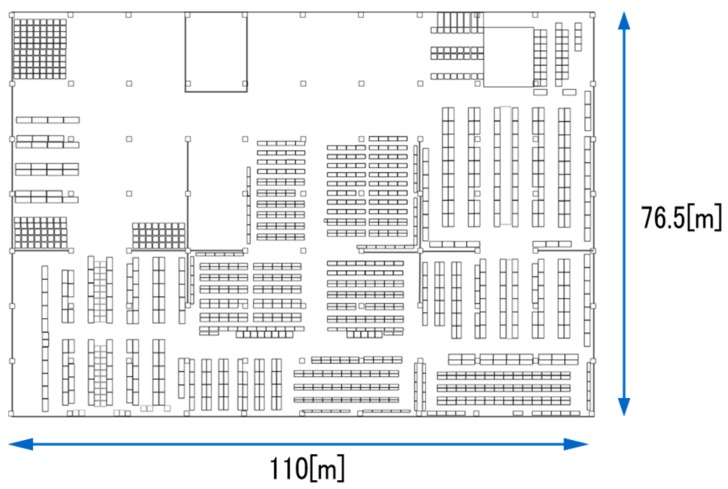
Detailed layout of the target warehouse that was provided to the participants.

Additionally, we shared the picking log data from the WMS. The WMS is often operated in warehouses for managing items and displaying work instructions for employees. In the case of a target warehouse, the barcodes are attached on each shelf, which are scanned with handy terminals for confirming items according to the WMS’s instructions. We utilized the logs of the barcode scan on each shelf as the ground truth of the employee positions. As subsidiary information about the warehouse, we shared a detailed layout of the warehouse (Figure 2), the correspondence table of the shelf identifications (IDs) and their locations, and the locations of the BLE beacons. 

**Figure 3:**
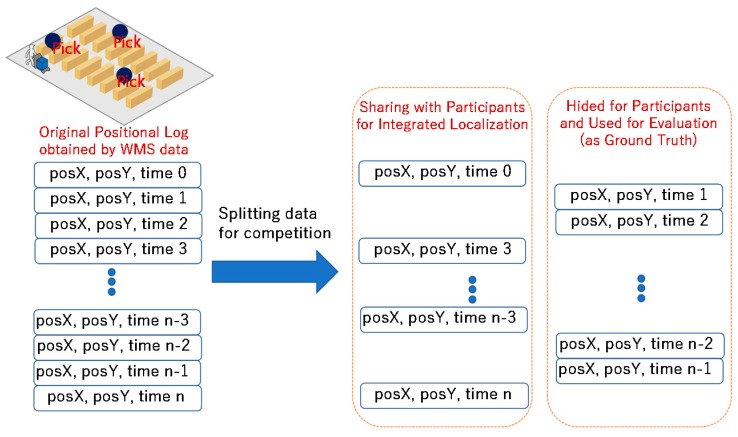
Positional logs extracted by warehouse management system (WMS) data are separated into two parts. One part is shared with the participants, who are supposed to use them for canceling the error in integrated localization. The other part is hidden for participants, and the organizer uses it as the ground truth for position evaluation.

Some parts of the ground truth of the locations from the WMS are shared for correcting the localization error. The remaining ground-truth data are hidden and utilized for evaluating the submitted trajectories from the competitors, as shown in Figure 3. The ratio of the hidden and unveiled ground truth can control the difficulty of integrated localization, because it can change the frequency of the availability of the unveiled ground truth. The competitors can utilize the WMS picking logs and RSSIs of BLE beacons and other information for improving the accuracy of employee locations. Therefore, this competition can be regarded as a competition for a PDR-based integrated localization method. 

### 3.2. Results of PDR Challenge 2017

#### 3.2.1. Evaluation Metrics for the PDR Challenge 2017

**Figure 4:**
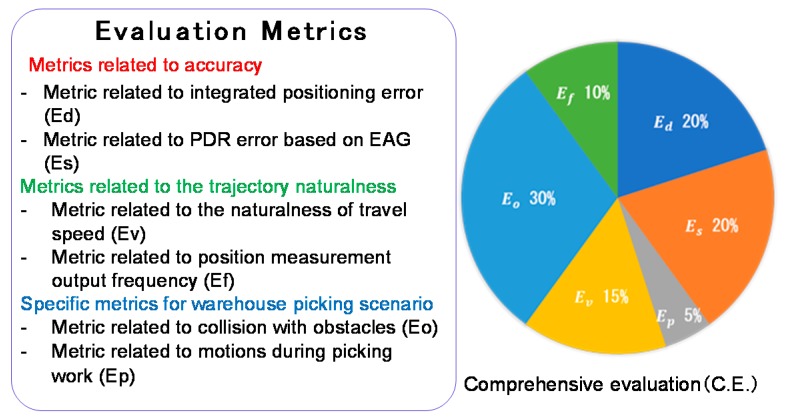
Submitted trajectories are supposed to be evaluated according to multi-faceted evaluation metrics. The final winner is determined by a comprehensive evaluation score (C.E.), which is calculated from the weighted sum of the elemental evaluation metrics.

The PDR Challenge 2017 aimed to be a valuable competition of PDR in a realistic situation. To evaluate the relative tracking method, relative accuracy, and error accumulation, factors other than the absolute accuracy need to be considered. The evaluation metrics of the PDR Challenge 2017 consist of not only the positional error, but also many possible evaluation metrics that are required for the warehouse scenario. In a realistic scenario, an ideal method should simultaneously meet comprehensive metrics such as positional error and obstacle interference metrics. We added subsidiary metrics, such as the frequency metric, for increasing the practicality of the submitted trajectories. According to the above concept, we defined the following six elemental evaluation metrics, which were categorized into metrics related to accuracy, trajectory naturalness, and special metrics for a warehouse picking scenario, as shown in Figure 4. The elemental metrics were weighted and summed for a comprehensive evaluation for reflecting all of the evaluation metrics. 

##### Evaluation Metric for Absolute Positional Error (E_d_)

**Figure 5:**
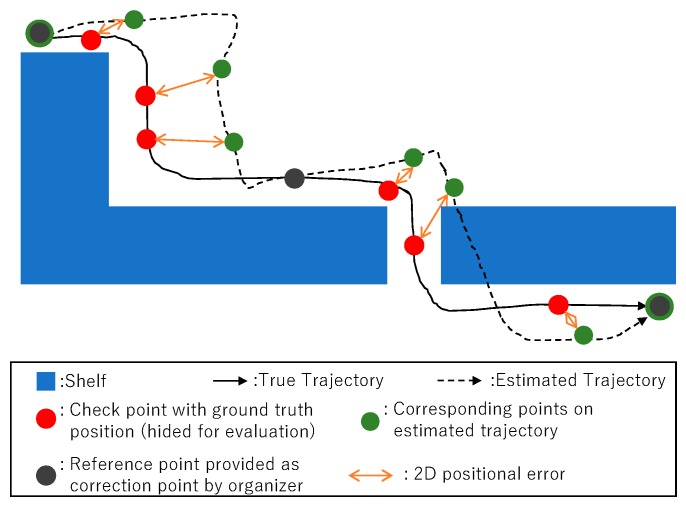
Evaluation metric for E_d_ is determined by calculating the distance between the ground-truth position of check points (colored in red) and corresponding points on an estimated trajectory (colored in green). The ground-truth data that are used for evaluation are discrete points obtained by the warehouse management system (WMS), and do not form a continuous path. We partially provide the ground-truth points (colored in gray) for correcting the error. At these points, the error can be canceled to 0.

This metric is an error metric of the absolute positional error. A conceptual image for the evaluation metric E_d_ is shown in Figure 5. The distance between the ground-truth position of the check points (colored in red) and the corresponding points on the estimated trajectory (colored in green) is calculated for determining E_d_. The ground-truth positions are extracted by the WMS, and are discrete points rather than a continuous path. Therefore, the evaluation of the absolute positional error is based on a discrete evaluation on the check points with the ground truth. The score for E_d_ is determined by calculating the median of 2D positional errors in the checking points. The target warehouse does not have multiple floors. We only consider the x and y coordinates of the position for evaluation by ignoring the height of the position. According to the naming style in the ISO/IEC 18305 standard, the median of the 2D positional error can be called CE50, which is derived from the 50th percentile value of the circular error. A detailed equation of E_d_ is shown in Appendix A.

##### Evaluation Metric for Error Accumulation of PDR (E_s_)

**Figure 6:**
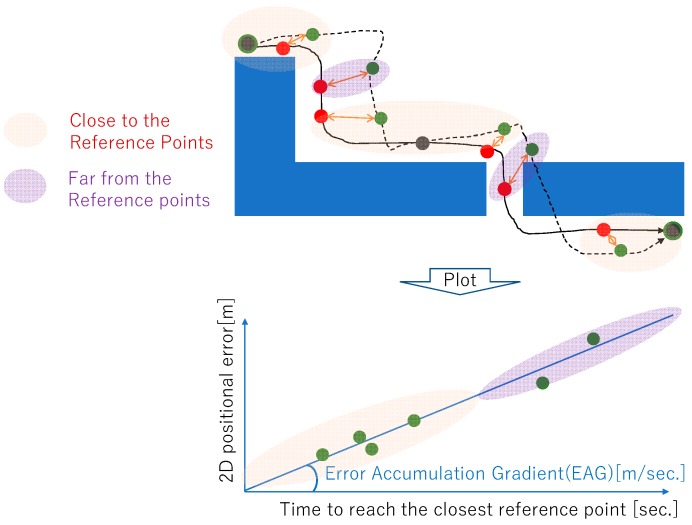
Score for the metric for the evaluation of error accumulation of PDR E_s_ is determined by calculating the speed of error accumulation. At the reference point, errors are assumed to be canceled to zero and to be increasing with time. The gradient of the error accumulation can be determined by plotting in the “time-error” space.

This metric indicates the speed of error accumulation caused by PDR. It is known that error accumulation is one of the main concerns for PDR. We partially provide the ground truth of the target’s positions for error correction. All of the competitors can correct the error to zero at the correction points. We assume that the error increases with time from the correction points, and that the error accumulation can be quantified as the speed of error accumulation from the correction points. As shown in Figure 6, taking inspiration from Abe et al. [15], we adopt linear regression between the time from the closest correction points and error in the “time-error” space. We assume the intercept of the linear model to be zero. A detailed equation of E_s_ is shown in Appendix A. We term the slope of error accumulation as the error accumulation gradient (EAG).

##### Evaluation for Naturalness During Picking (E_p_)

This metric is a dedicated metric for warehouse operation, and evaluates the naturalness of the trajectories during picking. As observed in the warehouse, employees stop in front of the shelves while picking items from them. Therefore, this should be reflected in the estimate at the time of picking. Using this metric, we check whether the target of the trajectories stops. More specifically, we check the length of the movement measured from 1.5 s before the picking to 1.5 s after the picking. If the movement is more than one meter, an index of this metric is deducted. The final E_p_ is calculated as the percentage of the checking points without the movement out of all the checking points. A detailed equation for E_p_ is shown in Appendix A. 

##### Evaluation of Velocity Naturalness (E_v_)

This metric checks whether the velocity in the trajectory is within the range of natural human movements. Some algorithm may be able to accurately estimate the position with a noticeable frequent jitter. We prefer a natural and smooth trajectory. To deduct such an artifact by a jitter, we check the velocity of all the submitted points. We define 1.5 m/s as the threshold of natural human speed, and calculate E_v_ is as the percentage of the checking points within the speed limit. A detailed equation for E_v_ is shown in Appendix A. 

##### Evaluation for Obstacle Interference (E_o_)

**Figure 7:**
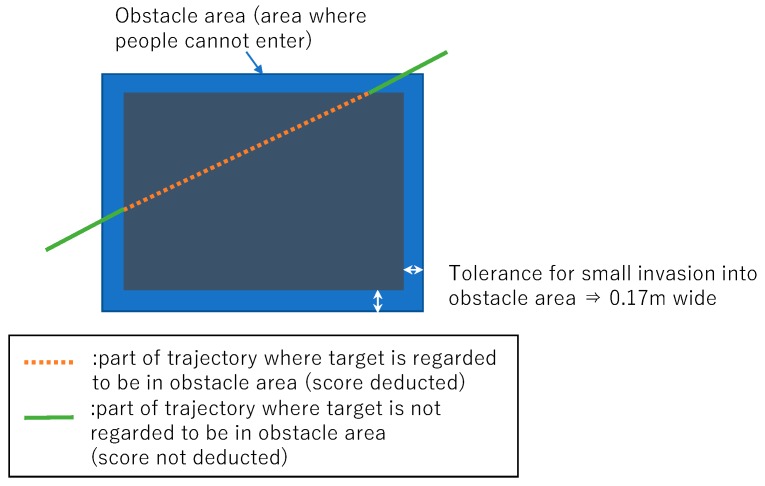
An evaluation metric for obstacle interference checks whether the points on the submitted trajectory enter the forbidden areas. This metric is evaluated at all of the points that comprise the trajectory. If the points lie in the obstacle area, the score is deducted. A 0.17-m wide tolerance area is defined for ignoring the small amount of invasion into the obstacle area.

This metric checks whether the trajectories enter the obstacle areas. We assume that ideal trajectories do not enter the area where employees cannot walk inside or pass over because of the existence of obstacles such as shelves, poles, and walls. The inconsistency between the trajectory and environments also affects the trajectory analysis, such as a traffic line analysis in warehouses. We introduce a metric E_o_ for quantifying the degree of incursion of the trajectories into the forbidden area, as shown in Figure 7. We share the location and size of the shelves, poles, and walls as reference information about the warehouse. Therefore, the competitors are informed about the obstacle areas. E_o_ is the ratio of the trajectory lengths that enter the obstacle area. A detailed equation for calculating E_o_ is shown in Appendix A. Note that we define a 0.17-m wide tolerance area around the borders of the obstacle area for ignoring a small amount of the incursion.

##### Evaluation for Update Frequency (E_f_)

This metric checks the update frequencies of the trajectories submitted by the competitors. If there are two trajectories with an accuracy that is the same as that of the positional estimation and different update frequencies, the trajectory with a higher frequency would have a greater value than the one with a lower frequency. Given the application of localization for the employees during the work, we define the minimum update frequency as one Hz. To calculate the update frequency for every frame, the elapsed time from the previously submitted frame is measured for all of the submitted frames. A detailed equation for calculating E_f_ is shown in Appendix A. 

##### Comprehensive Evaluation (C.E.)

To rank the submitted trajectories by quantifying the performance as an integrated indicator, the elemental evaluation metrics are integrated with the following weights. We term the integrated indicator as “comprehensive evaluation” (C.E.), which is calculated by Equation (1):

(1) $$C.E.=0.2E_{d}+0.2E_{s}+0.05E_{p}+0.15E_{v}+0.3E_{o}+0.1E_{f}$$

We believe that the error metrics (E_d_ and E_s_) and the metric for obstacle interference (E_o_) are more important than others, and thus, we set larger weights for them.

#### 3.2.2. Competitors and Their Algorithms

We announced the call for participation by releasing detailed information about the regulation, the measured data, and the target warehouse on the website for the PDR Challenge 2017 [16].

Consequently, we accepted applications from five teams [17,18,19,20,21].

According to the official regulation for all of the tracks of the IPIN 2017 competition, competitors were required to submit the technical descriptions about the algorithms and methods that they used for the competition. Here, the algorithms and methods of the competitors of our track are briefly introduced.

Team One’s method integrated inertial sensors, magnetic sensors, atmospheric pressure measurements, the WMS picking log, and the BLE signal logs by using attitude mixing, PDR, and the Kalman filter [17]. They use an activity classification algorithm for extracting the walking behavior from the complicated behavior observed in the actual picking operations. The Kalman filter using BLE and the WMS is used for maintaining the performance of the total integrated localization system.

Team Two’s method aimed to deal with the errors and difficulties caused by the different ways of holding the device and moving style [18]. Their algorithm focused on reducing the errors by position correction with the BLE signal from the beacons. For avoiding obstacle interference, their algorithm limited the movements of the targets only among the network graph, which was defined according to the warehouse map. They also consider influence of the obstacle in the BLE-based localization for the correction. They simulate the distribution of the BLE signals while considering the signal shielding by the obstacles. 

Team Three’s method utilized simulation for a human-like movement path, which avoided object interference by using the optimal reciprocal collision avoidance (ORCA) technique [19]. The simulated path was combined with movement estimation by PDR and BLE beacons for accurately estimating the final trajectory without obstacle interference. During the simulation, they divided the map into 0.3-m meshes, and classified each cell into a passible area or an obstacle area. In addition, they detect way points at which the worker seemed to stay very close to the BLE beacon. Such way points and WMS logs were used for optimal route calculation. Firstly, they determined the optical route between these intermediate points, and simulated the whole path using the network graph and ORCA technique. As the simulation result, a human-like trajectory could be obtained, which avoided the obstacle and other workers. A PDR algorithm by Ben et.al [22] was used for estimating the movement on the network graph, which labeled each move by step detection and move detection from the BLE. 

Team Four’s method utilized the zero velocity updates (ZUPT) algorithm with the Kalman filter (KF) for detecting the step length [20]. The moving direction was estimated using a gyroscope, and the positional error was corrected using a magnetic sensor and the MAP constraint. For obtaining step lengths, ZUPT with the inverted pendulum models was applied. Then, they were combined to estimate the trajectory. The KF was used for reducing the error of vertical movement. MAP and a magnetic sensor were subsidiarily used to guarantee the accuracy. In addition, an activity recognition was applied for detecting walking movement in the measured data.

Team Five’s method was based on the PDR algorithm, which estimated the steps by using an accelerometer and the moving direction by using an accelerometer and a magnetic sensor [21]. The WMS data were utilized for the starting and end points of each partial path between the WMS reference points. For removing the interference between obstacles and the estimated path, the partial path was shifted some amount when interference occurred. Their algorithm estimated the gravity vector by using the concept of a low-pass filter. Based on the gravity vector, they implemented a PDR algorithm with a step-heading system (SHS).

#### 3.2.3. Results of Evaluation

The submitted trajectories were evaluated using the evaluation metrics and the comprehensive metrics. In this section, we discuss the evaluation results. As a reference for the comparison, we add our result with our integrated localization method [23] based on our PDR algorithm [24].

Table 2 lists the results of all of the evaluation metrics and a comprehensive evaluation. As seen in the column of C.E., Team Two is ranked first in the table except for the AIST result. Team Two did not receive the highest scores in all of the elemental evaluation metrics. However, they received the highest score for metric E_o_, which had the highest weight. The competitors could calculate the evaluation metrics E_o_, E_v_, and E_f_ by themselves, because all of the information regarding the metrics was communicated to them. Team Two got almost perfect scores for those metrics, and thus seemed to have prepared their algorithm very well by tuning it for the regulations of PDR Challenge 2017. 

According to the technical descriptions, the algorithms of Team Two, Team Three, and Team Four contain efforts for avoiding interference by ORCA, a predefined network graph, or shifting. Team Two and Team Three got almost perfect scores for E_o_, and the E_o_ of Team Four was better than those of Team One and Team Five. Since E_o_ has the highest weight, the effort for avoiding object interference was a very important key for winning the competition. Another important fact is that most of the teams could successfully utilize the WMS log data, which is not common for the research community of indoor localization.

**Figure 8:**
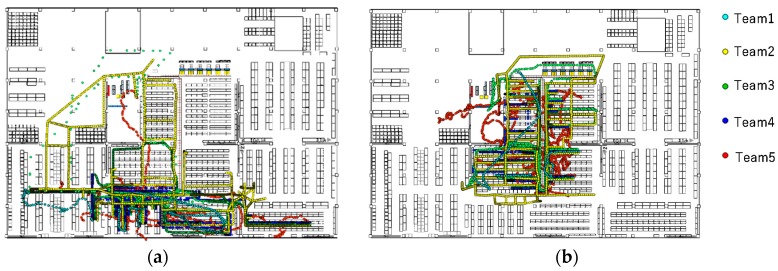
Example of submitted trajectories. (**a**) Trajectories for a sequence submitted by the competitors. (**b**) Trajectories for another sequence submitted by the competitions.

Examples of the comparison of the submitted trajectories are shown in Figure 8. The trajectories of Team Two are colored in yellow. As shown in the figure, Team Two’s trajectories consist of straight lines on the aisles in the warehouse. This is because their trajectories are generated with a predefined network graph. These trajectories do not look like natural human trajectories, but there is no specific metric in the regulation that deducts such trajectories. The deduction in E_p_ might be related to this, but it does not matter much, because the weight for *E_p_* is very small. 

**Figure 9:**
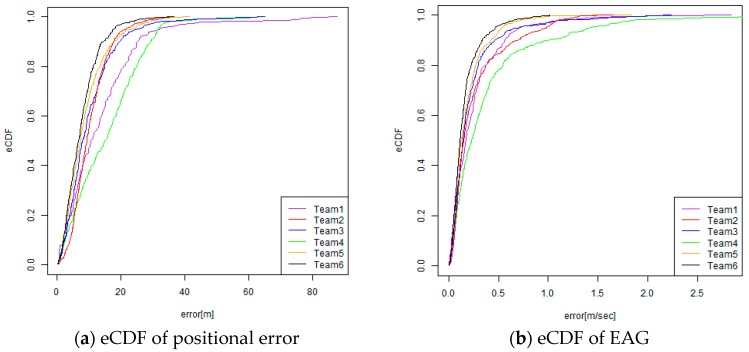
Empirical cumulative distribution function (eCDF) for positional error and error accumulation gradient (EAG).

One of the most important evaluation metrics is that for the absolute positional error, given by E_d_. Inspired by other competition tracks in IPIN [5,6,7], we also generated an empirical cumulative distribution function (eCDF) of absolute errors for understanding the error distribution. Figure 9 shows an example of eCDF for absolute positional errors. By using eCDF, we can easily calculate a 25th percentile error, 75th percentile error, and the median error, which is a 50th percentile error.

**Figure 10:**
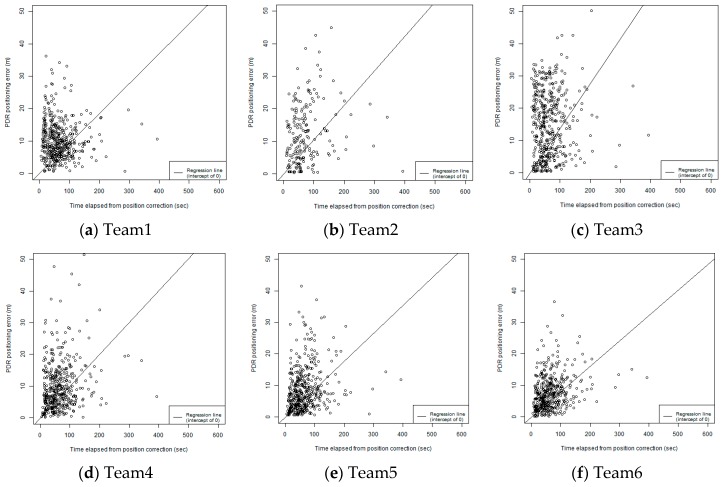
Comparison of slopes of error accumulation estimated by linear regression.

One of the unique characteristics of this competition is the adoption of the evaluation metric for the error accumulation of PDR as E_s_. As introduced in the previous section, E_s_ is proportional to the EAG, which is obtained by a simple linear regression in time-error space. Figure 10 shows a comparison of the slopes of error accumulation obtained by applying simple linear regression. As shown in the graph, there are many points that are regarded as outliers, which are the points far from the diagonal line. This result gives us an opportunity for discussing the application of simple linear regression for obtaining the EAG. However, we believe that this is a good starting point for quantifying the error accumulation for PDR. The EAG can be utilized for planning the infrastructure for integrated localization. For example, if the given EAG is equal to 0.1 m/s (six m/min) and the target accuracy of the localization is less than four meters, then the target accuracy can be accomplished if the positional corrections by an absolute positioning method with an error of less than one meter are available at least two times per minute (4.0 − 1.0 = 0.1 × 60/2).

According to the simulation, we can control the density of the arrangement of the infrastructure. Additionally, for the infrastructure whose sampling frequency is related to power consumption, the simulation result can be a rough indication for determining the setting of the update frequency.

### 3.3. Findings from PDR Challenge 2017

The results of the PDR Challenge 2017 introduced in the previous section can be summarized as follows:

We have successfully held a special tracking competition for warehouse picking by adopting multi-faceted evaluation metrics, which are required for evaluating the practicality of PDR-based localization methods in such scenarios.

We have successfully encouraged the competitors to develop practical localization methods that can fulfill the requirements for analyzing employees’ trajectories in a warehouse by adopting multi-faceted evaluation metrics.

The results of the scores calculated for all of the competitors indicate that the weight for E_o_ was too large. The winner of the competition performed “tuning” for this metric by adopting certain path-fitting algorithms instead of simply using the result of integrated localization. However, this tuning is completely fair, because the weights and regulations had been outlined in advance. To emphasize the naturalness of the PDR trajectories, the weight for E_o_ should be deducted. Moreover, other metrics and reference data for evaluating the naturalness of PDR trajectories are required.

We proposed the EAG for evaluating error accumulation. The EAG is not just an indicator for evaluation, but can also help in interpreting the physical meaning. More specifically, the EAG can quantify the degree of speed of error accumulation, which is one of the most important problems of PDR. The EAG can not only quantify the performance of PDR, but can also be utilized for planning the integrated localization and error correction by other absolute positioning methods. Particularly, the EAG can be used for helping a developer determine the sampling frequency of the correction methods and/or their density of arrangement. We adopted simple linear regression for obtaining the EAG. There is room for discussion regarding whether applying linear regression can accurately estimate the EAG.

### 3.4. Discussion of the Findings

As discussed in the previous sections, simple linear regression is easily affected by outliers. Additionally, we assumed that the competitors can propagate the partial ground truth in chronological order and reverse the chronological order. However, it is not guaranteed that all of the competitors can utilize the ground truth in such a manner. If a competitor propagates it only in a chronological manner, it increases errors even close to the correction points and further increases the EAG.

To investigate how to robustly estimate the EAG, we first test the linear regression with a robust estimation. We adopt the “rlm” function for a robust linear model fitting that is provided by the statistical computing language R, given that the intercept is zero. As other options, we can also generate the eCDF for the EAG. The EAG can be calculated for each checking point. Figure 9b shows an eCDF of EAG for all of the checking points in all of the trajectories. By referring to the graph of eCDF, we can easily calculate the 25th percentile of the EAGs; the 50th percentile of the EAGs, which is equal to the median of the EAGs; and the 75th percentile of EAGs. Inspired by the naming style of ISO/IEC 18305, we call them EAG25, EAG 50, and EAG75, respectively. Table 3 compares the methods for estimating the EAG in the original linear regression, the linear regression with robust estimation, and EAG25, EAG50, and EAG75. 

As seen in Table 3, the first ranked team in terms of the EAG is always AIST, and the second ranked is Team Three; however, the lower ranked teams vary in the methods. It is impossible to determine the best methods from these results alone. However, it seems to be better to check and compare the results from multiple methods for better understanding error accumulation.

During the exchange of e-mails with competitors and question and answer session (Q&A) of the special session of competitions at IPIN 2017, we received fruitful feedback for improving the competition and its evaluation. We summarize the feedback, including the self-review, as follows. 

A team requested that we provide them with sample videos of the recording of the picking work in the warehouse, because they were not familiar with the warehouse operation.

We have not implemented a fully automated system for sharing and receiving the data as well as analyzing the submitted trajectories. It was possible to deal with five contestants, but the automated system is required for facilitating the organizers’ work.

There are some employees driving forklifts rather than walking during the operation. The PDR Challenge did not deal with the data of employees who drove the forklift, because the methodology for tracking a forklift is different from that for tracking a pedestrian. Tracking a forklift has been added because the tracking of targets is required for future competitions.

## 4. xDR Challenge for Warehouse Operations

### 4.1. Overview of the Competition

**Figure 11:**
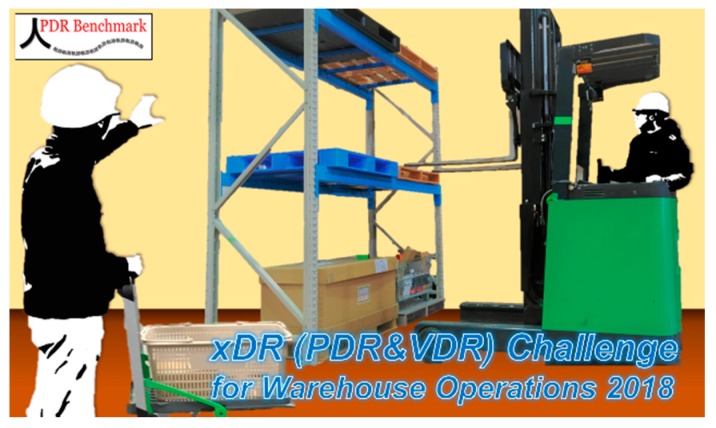
Teaser image of the xDR Challenge that were used for the call for participation.

As described in Section 2.4, the xDR Challenge 2018 was held as a sequel off-site competition of the PDR Challenge series. Similar to the PDR Challenge 2017, the scenario of the xDR Challenge 2018 was a behavior analysis of the employees working in warehouses. The update of the xDR Challenge 2018 from the PDR Challenge 2017 was an addition of the estimation of the trajectory of forklifts driven by employees as a tracking target. The vehicle dead-reckoning (VDR) is assumed to be used for tracking forklifts. The xDR Challenge 2018 was regarded as the world’s first competition that deals with VDR. The evaluation methodologies adhere to the PDR Challenge 2017. Thus, the winner of the competition is determined using the integrated evaluation metric. Beyond that, the scale of the data in terms of the number of employees and the length of the measurement was expanded, and additional information was provided according to the findings from the PDR Challenge 2017. Figure 11 shows the conceptual images that were used for the call for participation. 

### 4.2. Data Used for Xdr Challenge 2018

As described earlier, the xDR Challenge 2018 was an off-site competition that utilized the data measured during actual warehouse operations in a real warehouse. In this section, the data used for the competition are introduced.

The test data were measured for a week in March 2018. We asked 34 employees to participate in the measurements for the PDR test data. Six forklifts were also measured for VDR test data.

In total, we could collect about 170 sequences for PDR and about 30 sequences for VDR.

The sensor data that was used as the test data for PDR and VDR were measured in the same manner as that for the sensor data measurement for the PDR Challenge 2017. We utilized Android smartphones with the sensor data logging application. The application collected the angular velocity, acceleration, magnetism, atmospheric pressure, and BLE signal from the BLE tags at 100 Hz.

Similar to the PDR Challenge 2017, we asked the employees to operate the application by themselves. The operations that we asked included starting a logging application by inputting the employee code and finishing the logging application. The sensor data for VDR were measured using a similar Android application. The smartphones for VDR were taped to the forklifts.

The usage of the WMS data was also the same as that of the PDR Challenge 2017. We used the WMS logs for evaluating the submitted results and providing a partial reference for canceling the integrated localization error at specific points in the measurement. As observed in the warehouse, employees who move by foot stop in front of the shelves when they pick items from the shelves. An order picking fork is a type of forklift that can directly lift the driving base and thus allow the driver to pick the items from high shelves by hand. Although several types of forklifts are operated in the target warehouse, we pick up the data measured with the order picking fork for the competition. Therefore, we can assume that forklifts also stop in front of the shelves when the drivers pick items from the shelves. In addition, subsidiary BLE tags are mounted on the forklift for detecting accesses to the forklifts and utilized for filtering the WMS logs. Owing to this, in the VDR track, we can utilize WMS data in the same manner with the PDR track.

Although the total amount of data was huge, as described above, we carefully selected the data according to the consideration of the data size and the distribution of WMS logs that were provided to the participants as the test data. The statistics of the test data are shown in Table 4.

Similar to the PDR Challenge, we also provided sample data before accepting the final application to the competition. The participants could test the algorithm with sample data in order to determine the final decision of the participation. As a new trial, we added a computer graphics (CG) animation of the picking operation for introducing what the picking operation looks like. For creating the animation, the whole-body motion during the picking operation was captured by a representative worker for two hours with an inertial measurement unit (IMU)-based motion capture system, which was named Awinda by Xsens.

### 4.3. Evaluation Method

#### 4.3.1. Evaluation Metrics

The evaluation method for the xDR Challenge 2018 basically adhered to the PDR Challenge 2017, which means that the final scores were calculated by multi-faceted metrics, including a dedicated evaluation PDR and a warehouse scenario. The evaluation metrics were renamed for clarifying the meaning of each metric. The evaluation metrics can be categorized into six elemental metrics: evaluation for accuracy of trajectory, naturalness of trajectory, and special evaluations for a warehouse scenario, which were the same as those for the PDR Challenge 2017.

- Evaluation for accuracy of localization (modified from PDR Challenge 2017)
  ○ E_median_error_: Evaluation of absolute amount of error of integrated localization
  ○ E_accum_error_: Evaluation of speed of error accumulation of PDR/VDR
- Evaluation for naturalness of trajectory (same as PDR Challenge 2017)
  ○ E_velocity_: Evaluation of speed assumed for the target
  ○ E_frequency_: Evaluation of frequency of points comprising the trajectory
- Special evaluations for warehouse scenario (same as PDR Challenge 2017)
  ○ E_obstacle_: Metric related to collision with obstacles
  ○ E_picking_: Metric related to motions during picking work

#### 4.3.2. Updates of Evaluation Metrics from the PDR Challenge 2017

The updates of the evaluation metrics from the PDR Challenge are introduced as follows:

● Updates of the method for calculating the representative EAG.

The EAG was calculated at a stage prior to calculating the E_accum_error_ (which was called E_s_ in the PDR Challenge 2017). The EAG is an indicator that quantifies the speed of error accumulation. In the xDR Challenge 2018, we changed the way of calculating the representative EAG. In the PDR Challenge, the speed of error accumulation was quantified by calculating the slope of the linear function estimated by simple linear regression. In the xDR Challenge, first, we generated the eCDF of the EAG. Then, the median, which is equal to the EAG50, was regarded as a representative EAG.

● Weights for calculating integrated evaluation metrics are modified.

As discussed in Section 3.2.3, the weight for E_o_ was relatively high at the PDR Challenge 2017, because we acknowledge the significance of special evaluation for a warehouse scenario. However, in the xDR Challenge 2018, we worried about over-tuning this metric by ignoring the metrics of accuracy. Thus, we increased the weights for evaluating the metrics of accuracy while reducing the weights for E_obstacle_. The final weights that were used for the competition are shown as follows:

E_median_error_:E_accum_error_:E_velocity_:E_frequency_:E_obstacle_:E_picking_=30%:30%:10%:10%:15%:5%

● Modification of evaluation of PDR error accumulation

In the PDR Challenge 2017, the evaluation metric E_s_ evaluates the speed of PDR error accumulation from the reference points by WMS. However, the BLE signal information was also included in the sensor data of the target periods of PDR error accumulation. Therefore, the evaluation was not the evaluation of trajectory by pure PDR, but that of integrated localization.

**Figure 12:**
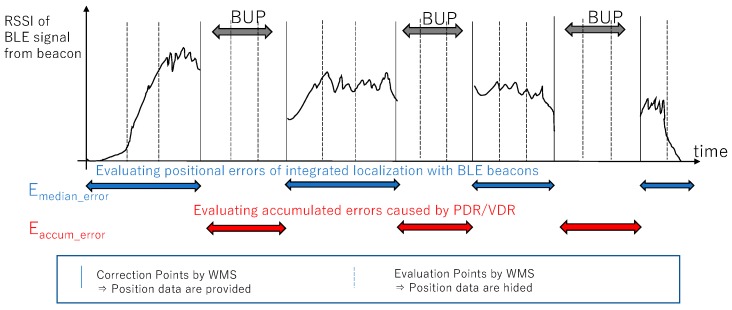
Evaluation of error accumulation caused by PDR/VDR with a BLE unreachable period (BUPs).

**Figure 13:**
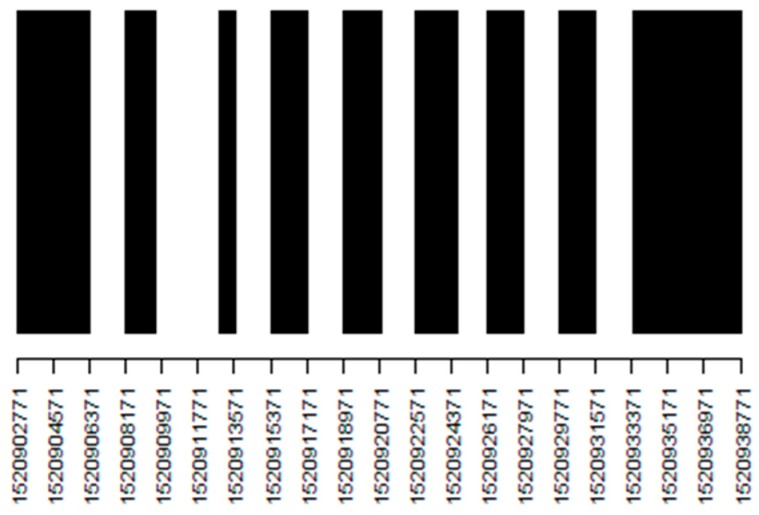
An example of BUPs defined for a test data.

In the xDR Challenge 2018, we adopted the period in which BLE signals were intentionally erased from the sensor data. We defined these periods as the BLE unreachable period (BUP), which is conceptually illustrated in Figure 12. BUPs are defined between the reference points provided by the WMS, because at these reference points, the errors can be cancelled to zero. As shown in Figure 12, we evaluate for the E_accum_error_ in BUPs and E_median_error_ out of BUPs. An example of the BUPs that were defined for the test data is shown in Figure 13. In the figure, the BUPs and non-BUPs are colored in white and black, respectively. They are evenly distributed, and are switched around approximately every 30 min. The actual intervals of BUPs are varied according to the distribution of the reference data by the WMS. 

### 4.4. Competitors and Their Algorithms

We announced the call for participation for the competition by releasing the detailed information of the regulation and information about the measured data and target warehouse on the website for the xDR Challenge 2018 [25].

In this section, the competitors and their algorithms are briefly introduced in the same manner as that for the PDR Challenge 2017, as described in Section 3.2.3. In the xDR Challenge 2018, we accepted seven teams as pre-registrants for obtaining the sample data. Finally, five teams joined the PDR track, and two joined the VDR track. However, a registrant who registered for both tracks withdrew at the last stage during the test period. 

The xDR Challenge 2018 was not an official competition track of IPIN 2018; however, the special session was held for announcing the results of the competition. Therefore, publications describing the technology that was used for the competition were not published from the IPIN 2018 or IEEE. To grasp the technologies that were used for the competition, we asked the participants to submit a short description about the algorithms or methods. One team submitted the results, but they did not submit the technical description. Therefore, in the remainder of this paper, the results from the three teams are evaluated as the results from the final registrants. The algorithms from the three teams are introduces as follows.

Team One’s method was an integrated localization method with the BLE, the WMS log, and the PDR/VDR method with a drift correction of the gyro sensor by detecting a straight line and the stopping period in the trajectory. The relative movement calculation was based on the existing PDR method [24] for the PDR and the VDR methods [14] for VDR. They integrated the result of the relative movement by PDR and VDR, the result of the BLE-based localization, and the WMS logs. In the integrated localization, they adopted a novel discrete state-space model for detecting the staying area and its transition with the grid segmentation of the target area for estimating the movements between the reference points that were provided as the WMS data. The transition in the grid segmentation was limited only in the passage areas. Thus, map matching was realized. A well-known smoothing algorithm was used for the smooth state estimation.

Team Two’s method was a PDR method with step detection by an accelerometer, moving direction estimation by an accelerometer and a gyroscope, and position and direction correction with BLE beacons. More specifically, the moving distance was calculated by analyzing every step that was calculated by using acceleration. Also, the posture of the smartphones was calculated by using the acceleration and angler velocity. The moving directions of the workers were estimated by the BLE data. The BLE data were also used for correcting the absolute position. These estimations were combined for generating the final trajectories.

Team Three’s method is an integrated localization method based on the SHS-type PDR with angular drift correction by utilizing the straight lines in MAP. They utilized the WMS and map matching for position correction. More specifically, they estimated the trajectory of PDR by using algorithms for step counting, step length, and heading direction estimation. Then, drift errors over a long period of time were corrected. In this correction, they utilized that most of the aisles were either horizontal or vertical. Finally, they applied postprocessing using constraints by the WMS and MAP.

### 4.5. Evaluation Results

The final scores for all of the evaluation metrics are shown in Table 5 and Table 6. Besides the score, as important indicators calculated for the scores, CE50 and EAG50 were also introduced in the same table. Note that the scores are calculated for individual trajectories and the calculating average for filling this table. CE50 means the 50th percentile of circular error, which is an error in the 2D plane compared to the ground-truth defined in the 2D coordinate; meanwhile, C.E. means the comprehensive evaluation, and is the score used for determining the winner.

As the result, Team One won the PDR tracks of the competition, and Team Three was the runner-up. In the VDR track, there was only one participant. As shown in Table 5, the top two teams obtained evenly good scores for all of the evaluation metrics. Thus, all of the evaluation metrics seemed to contribute to determining the winners. 

As shown in Table 6, the results in the VDR track were worse than those for the PDR track. This might be by the reason that the relative tracking by VDR was not as mature as that by PDR at this moment. Another possible reason is that the accuracy of the ground truth obtained by the WMS for the VDR track. We selected the WMS picking logs that seemed likely to be picked from very close to the forklifts by using the subsidiary BLE tags mounted on the forklifts. The error of the true position of the forklifts and the shelves might have caused the decrease of the score for the error evaluation for the VDR track.

Team One adopted the grid-segmented transition model. This can be regarded as a type of transition on a very dense network graph. Owing to this effort, they obtained a perfect score for the E_obstacle_ value. Team One and Team Three also obtained almost perfect scores for this metric. One of the characteristics in common with those of the high achievers was the adaptation of the drift correction method. This year, we did not provide calibration data that recorded the data at rest. Therefore, the dynamic calibration method is very important for canceling the drift error. Their algorithm tried to detect straight lines or rest stages for such purposes. Dynamic and online calibrations are important for maintaining accurate localization for long-term measurements.

**Figure 14:**
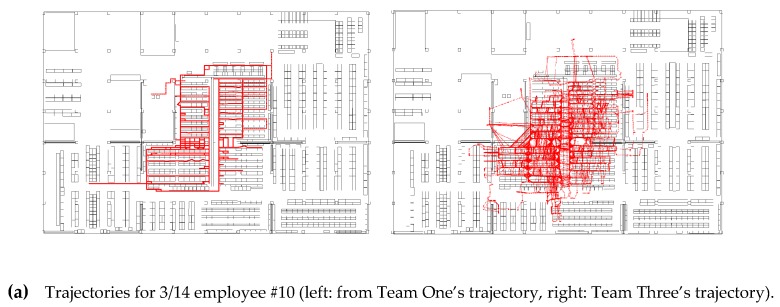

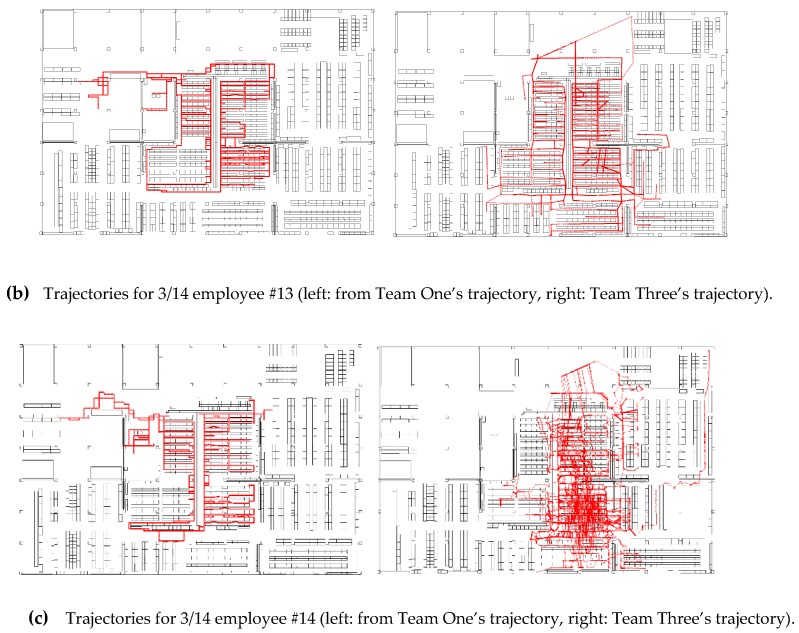
Examples of trajectories in the PDR track submitted by high achievers.

**Figure 15:**
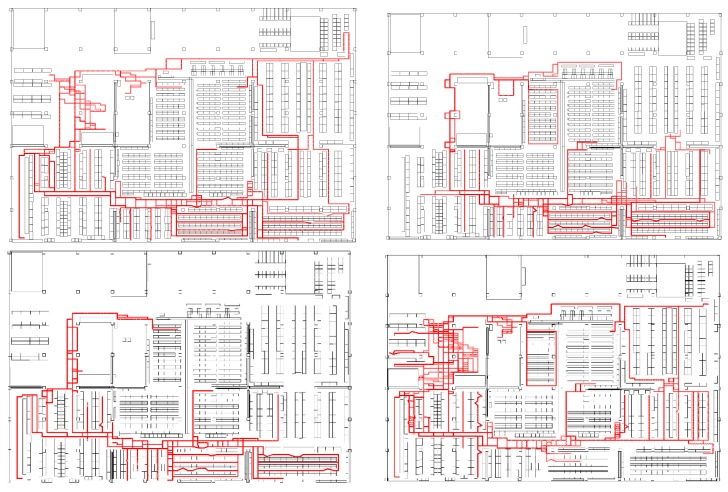
Examples of trajectories in the VDR track (**Top Left**: 3/15 Dev#57, **Top Right**: 3/19 Dev#57, **Bottom Left**: 3/14 Dev#13, **Bottom Right**: 3/19 Dev #5).

Figure 14 and Figure 15 show the examples of submitted results from the participants for the PDR and VDR tracks. As shown in the figures, most of the participants could estimate the positions of the targets by localization methods based on PDR or VDR during the BUPs when the BLE signals were intentionally deleted. In Figure 14, the efforts for avoiding the obstacle interference and online calibration using straight lines can be seen. Most of the line directions were horizontal and vertical, and trajectories existed mainly in the aisles of the warehouse. In Figure 15, it can be seen that the forklifts were driven only on the wide aisles that were mainly located on the outer area. Such trajectory types can be seen in the submitted results on the VDR tracks.

**Figure 16:**
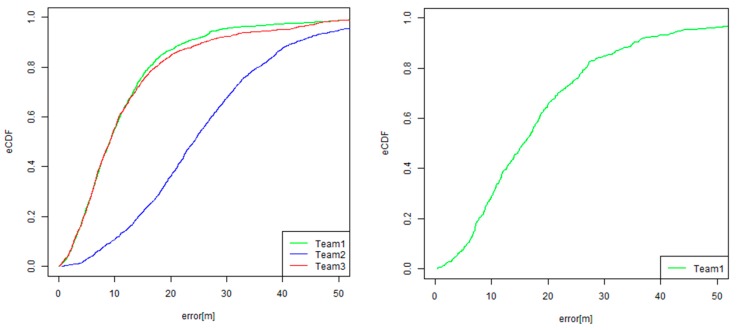
Results of the eCDF of the C.E. (circular error) (**Left**: PDR track, **Right**: VDR track).

**Figure 17:**
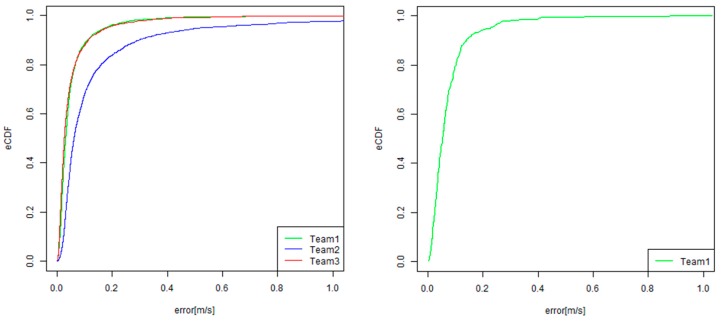
Results of the eCDF of the EAG (**Left**: PDR track, **Right**: VDR track).

Figure 16 and Figure 17 show the graphs of the results of the eCDF of the CE and the EAG. These graphs also indicate the top two teams that went head-to-head in the competition regarding both of the E_median_error_ and E_accum_error_ values. It was also found that the difference of the CE between the two teams (green and red) increased between from the 60th percentile and the 95th percentile. Therefore, it seems that Team One (green) could successfully reduce the error for not only the CE50, but also for the CE in the worse cases.

Table 7 shows the comparison of CE50 and EAG50 between the in/out values of the BUP. As expected, all of the CE50 and EAG50 values in the BUP were larger than those of the corresponding results of “out of BUP”. This indicates that all of the participants could successfully utilize the records of signals from the BLE tags in “out of the BUP”. The difference of the CE50 and EAG50 values between the in/out BUP values varied from one team to another. The degree of decrease might be related to the degrees of dependence on the BLE beacons. The decrease in the CE50 and EAG50 values for Team One was relatively high compared to the other teams. This indicates that Team One’s degree of dependence on the BLE was high. In addition, Team Two and Team Three could also utilize the BLE information, but the contributions of the BLE tags were little. This finding is a by-product of the evaluation with BUP, and was not considered for the final comprehensive evaluation (C.E.). However, this is quite an interesting finding, because the elements of the integrated localization system can be decomposed for further understanding the system. 

Unfortunately, there was only one participant in the VDR track. The VDR technology itself has a low profile at this moment, and the barrier to entry seemed to be still high. This competition might be an enlightenment activity, but further enlightenments of the VDR technology are required. 

## 5. Releasing the Evaluation Program Used for PDR Challenge and Xdr Challenge Via Github

After the PDR Challenge 2017 and xDR Challenge 2018, we received inquiries about the disclosure of the ground truth obtained by the WMS and the evaluation program. It is difficult to widely release the ground-truth data, because the WMS data are supposed to be limitedly provided for the competition’s participants under the agreement between the warehouse owner and the competition organizer. On the other hand, the evaluation frameworks and the philosophy of the evaluation in the competitions are expected to be widely spread. Therefore, we decided to release an evaluation program through the Internet. The evaluation program is expected to be refined by obtaining feedback from the users. From the standpoint of the participants, continuous refinement of the algorithm after the result submission can be facilitated by enabling the feedback loop with the evaluation tools and ground truth. 

As a platform for the disclosure of the evaluation program, we adopted GiHub [26]. To check and modify the program details, we also uploaded the source code of the evaluation program. Currently, we have only uploaded the exactly same evaluation program as that used for the xDR Challenge 2018, especially for “after-competition services” for participants [27]. We are currently working on generalizing the evaluation method and simplifying the evaluation procedure for the program. The supporting tool for the preparation of the required files for the evaluation and detailed documents are also required for spreading the evaluation framework.

## 6. Conclusions

In this paper, we introduced indoor localization competitions organized by us, which were named the “PDR Challenge in Warehouse Picking 2017” and the “xDR Challenge for Warehouse Operation 2018.” These competitions were held under the warehouse scenario, which is one of the most remarkable industries that require improvement in efficiency. The main characteristics of our competitions were clarified by surveying the existing indoor localization competitions. Our competitions are very realistic, because their test data were measured during operations in an actual warehouse. In addition, the evaluations comprised not only the evaluations of accuracy of localization, but also the evaluations of the other performances that are required for PDR/VDR and a warehouse-dedicated scenario. Additionally, the VDR track of the xDR Challenge is the world-first competition for tracking forklifts by VDR.

One of the most important evaluations in our competitions was the evaluation of the accumulation of error caused by the nature of relative tracking with PDR or VDR. We named and quantified the error accumulation by EAG, which corresponds to the speed of error accumulation. EAG can be utilized for the preliminary consideration of the indoor localization system in terms of the estimation of the accuracy and planning of the correction methods for achieving the target accuracy. In the xDR Challenge 2018, we refined the method for evaluation with EAG by adopting the BUP for the evaluation of pure effects from PDR or VDR.

Besides the estimated trajectory, we could collect the technical descriptions regarding the methods used for the competitions, and thus could analyze the relation between the algorithms and the results. The characteristics of the methods that the high achievers of the PDR Challenge 2017 had in common included the adaptation of drastic methods for obstacle interference, such as network graph-based trajectory generation or ORCA. These methods might decrease the accuracy of absolute error. Considering the high weight of the obstacle interference for comprehensive evaluation, it was quite a good strategy. In the xDR Challenge 2018, the evaluation method was refined and the type of targets and scale of the measurement were expanded by reflecting on the feedback of the PDR Challenge 2017. The xDR Challenge 2018 also attracted many participants from various counties, and was completed successfully. In terms of the evaluation metrics, the refinement of the evaluations worked very well, because high achievers obtained evenly good scores for all of the metrics, which means that all of the evaluation metrics could not be ignored for winning the competition. The trend of the high achiever’s algorithm was the adaptation of an online or dynamic calibration for correcting the drift error. These techniques in trend were a very hot topic and very crucial, especially for this competition, because no static calibration data were provided. Unfortunately, the number of participants in the VDR track was small; thus, further enlightenment of the xDR Challenge and VDR technology itself is required. 

As future work, we will keep holding the PDR Challenge series competitions by enriching the contents of the competition, as well as promoting the VDR track. In particular, we plan to spread the competition beyond the logistics industries, such as to the manufacturing industries and restaurant business. In addition, we plan to expand the target technologies that are dealt with in the competition. For example, current plans for targets include whole-body posture measurements and behavior recognitions, which both have strong a relation with indoor localization. We would like to differentiate our competitions from other competitions as an ambiguous competition series that is mainly for industry by challenging important topics in the industry.

## Figures and Tables

**Table 1:** Brief survey of existing indoor localization competitions.

	PerfLoc by NIST	EvAAL /IPIN Competitions	Microsoft’s Competition@IPSN	Ubicomp/ISWC 2015 PDR Challenge	PDR Challenge in Warehouse Picking in IPIN 2017	xDR Challenge for Warehouse Operations 2018
**Scenario**	30 Scenarios (Emergency scenario)	Smart House/Assisted Living	Competing maximum accuracy in 2D or 3D	Indoor pedestrian navigation	Picking work inside a logistics warehouse (Specific Industrial Scenario)	General warehouse operations including picking, shipping and driving forklift
**Walking/Motion**	walking/running/ backwards/sidestep/ crawling/pushcart/ elevators (walked by actors on planed path with CPs)	Walking/Stairs/Lift/Phoning /Lateral movement (walked by actors on planed path with CPs)	Depends on operators (developers can operate their devices by themselves	Continuous walking while holding smartphone and looking at navigation screen	Includes many motions involved in picking work, not only walking	Includes many motions involved in picking, shipping operations and, not only walking. Some workers may drive forklift
**Tester**	Tester assigned by the organizer	Tester assigned by the organizer	Participants can run their system by themselves	Conference participants	Employees working in the warehouse	Employees working in the warehouse
**One-site or Off-site**	Off-site competition and Live demo	Separated on-site and off-site tracks	On-site	Data collection: on-site Evaluation: off-site	Off-site	Off-site
**Target Methods**	Arm-mounted smartphone based localization method (IMU, WiFi, GPS, Cellular)	Off-site: Smartphone base On-site: Smartphone based/ any body-mounted device (separated tracks)	2D:Infra-free methods 3D:Allowed to arrange Infra. (# of anchor and type of devices are limited in 2018)	PDR+MAP	PDR+BLE+MAP+WMS	PDR/VDR+BLE+MAP+WMS
**# of people and trial**	1 person × 4 devices (at the same time) × 30 scenario	Depends on year and track (e.g. 9 trials, 2016T3)	N/A	90 people, 229 trials	8 people, 8 trials	34 people + 6 forklifts, 170 trials (PDR) + 30 trials (VDR)
**Time per trial**	Total 16 hours	Depends on years and tracks (e.g. 15 mins (2016T1,T2), 2 hours (2016T3))	N/A	A few minutes	About 3 hours	About 8 hours
**Area of test environment/** **length of test path**	30,000 m^2^	Depends on years and tracks (e.g., 7000 m^2^ @2016T1&T2)	Depends on years (e.g., 600 m^2^ @ 2018)	100 m each	8000 m^2^	8000 m^2^
**Evaluation metric**	SE95 (95% Spherical Error)	75 Percentile Error	Mean error	Mean error, SD of error	Integrated Evaluation (integrated by accuracy, naturalness, warehouse dedicated metrics)	Integrated Evaluation (integrated by accuracy, naturalness, warehouse dedicated metrics)
**History**/**Remarks**	1 time (2017–2018)	7 times (2011, 2012, 2013, (EvAAL), 2014, 2015 (+ETRI), 2016, 2017 (EvAAL/IPIN))	5 times (2014, 2015, 2016, 2017, 2018)	Collection of data of participants walking. The data are available at HASC (http://hub.hasc.jp/) as corpus data	Competition over integrated position using not only PDR, but also correction information such as BLE beacon signal, picking log (WMS), and maps	Consists of PDR and VDR tracks. Referential motion captured by MoCap. also shared for introducing typical motions.
**# of Participants**	169 (registration)/16 (submission)	Depends on years and tracks (T1:5,T2:12, T3:17:T4:5@2018)	Depends on years and tracks (2D:12,3D:22@Y2018)	4 (+1 Unofficial submission)	5	PDR:5, VDR:2
**Awards and Prizes for 1st Place**	US$20,000 (US related group only)	Depends on years and tracks (e.g. 1500€ @2016)	US$1000	JP\50,000	JP\100,000 + prize or JP\150,000	JP\200,000 (or JP\150,000) + prize(s)
**Connection with Academic Conference**	N/A	IPIN	IPSN	UbiComp/ ISWC2015	IPIN2017(an official competition track	IPIN (Special Session)

**Table 2:** Results of evaluations in the PDR Challenge 2017. C.E: comprehensive evaluation score, EAG: error accumulation gradient.

Team	E_d_	E_s_	E_p_	E_v_	E_o_	E_f_	CE50 (Ref)	EAG50 (Ref)	C.E.
Team 1	66.876	93.692	97.195	99.998	51.821	11.323	10.606	0.173	68.652
Team 2	71.524	94.872	43.545	100	99.876	100	9.258	0.150	90.419
Team 3	76.459	95.333	72.719	87.835	93.549	99.271	7.827	0.141	89.161
Team 4	51.934	90.769	84.965	95.657	59.623	99.239	14.939	0.230	74.948
Team 5	78.386	96.308	97.484	99.093	45.530	100	7.268	0.122	78.336
Team 6 (AIST)	80.272	96.718	81.057	98.711	89.968	95.879	6.721	0.114	90.836

**Table 3:** Comparison of methods for determining the representative EAG.

Team	EAG by Simple Linear Regression	EAG by Robust Linear Regression	EAG25	EAG50	EAG75
Team 1	0.106	0.128	0.069	0.173	0.311
Team 2	0.139	0.169	0.095	0.230	0.441
Team 3	0.089	0.100	0.064	0.122	0.240
Team 4	0.100	0.107	0.077	0.141	0.262
Team 5	0.093	0.106	0.085	0.150	0.309
Team 6 (AIST)	0.081	0.087	0.062	0.114	0.186

**Table 4:** Statistics of test data for the xDR Challenge. VDR: vehicle dead-reckoning.

Tracks	# of Trajectories	Total Time Length of Test Data	# of WMS Points Provided	# of WMS Points Hidden for Evaluation
PDR	15	180 hours	271	4877
VDR	8	85 hours	125	1027

**Table 5:** Final scores for the evaluation of the xDR Challenge 2018 (PDR track).

PDR Track	E_median_error_	CE50	E_accum_ error_	EAG50	E_velocity_	E_frequency_	E_obstacle_	E_picking_	C.E
Team 1	72.14	9.08	100	0.031	99.00	99.87	100	98.73	91.47
Team 2	20.36	24.01	99.44	0.061	99.00	79.06	99.93	99.00	73.68
Team 3	71.99	9.12	100	0.026	96.93	100.00	97.60	95.80	90.72

**Table 6:** Final scores for the evaluation of the xDR Challenge 2018 (VDR track).

VDR Track	E_median_error_	CE50	E_accum_error_	EAG50	E_velocity_	E_frequency_	E_obstacle_	E_picking_	C.E
Team 1	49.30	15.70	99.86	0.053	99.75	99.25	100	97.38	84.51

**Table 7:** Comparison of the CE50 and EAG50 values in/out of BUP.

Track and Team	CE50 in BUP	CE50 Out of BUP	CE50 in Whole traj.	EAG50 in BUP	EAG50 Out of BUP	EAG50 in Whole traj.
PDR: Team 1	11.28	9.08	10.17	0.031	0.027	0.029
PDR: Team 2	25.33	24.10	24.70	0.061	0.064	0.063
PDR: Team 3	9.74	9.12	9.48	0.026	0.025	0.026
VDR: Team 1	18.45	15.70	16.35	0.053	0.044	0.048

## Appendix A

The detailed equations for calculating the evaluation metrics are as follows:

Score of metric for integrated positioning error: E_d_

$$\begin{array}{cc} E_{d}=100 & \;(\mathrm{CE}50\;<\;1)\\ E_{d}=100-\frac{100}{29}\left(CE50-1\right) & \;(30>\mathrm{CE}50\;>\;1)\\ E_{d}=0 & \;(\mathrm{CE}50>\;30), \end{array}$$

where the median of 2D positional errors is CE50.

Score of metric for PDR error based on EAG: E_s_

$$\begin{array}{cc} E_{s}=100 & \;(\mathrm{EAG}\;<\;0.05)\\ E_{s}=100-\frac{100}{1.95}\left(EAG-0.05\right) & \;\;(2.0>\;\mathrm{EAG}>\;0.05)\\ E_{s}=0 & \;(\mathrm{EAG}>\;2.0){,}_{\;} \end{array}$$

where the slope of linear regression of errors is EAG.

Score of metric for motions during picking work: E_p_

Given, $Flag_{i}^{p}$ is assigned one when the target stops, and zero when the target does not stop at the *i*th checking points.

$$E_{p}=\frac{\sum_{i=1}^{N_{pick}}Flag_{i}^{p}}{N_{pick}}\times 100,$$

where N_pick_ is the number of all the checking points.

Whether the target stops or not is determined by length of partial trajectory $Move_{i}^{p}$ between from the 1.5 s before the *i*th checking points and 1.5 s after the *i*th checking points. If the $Move_{i}^{p}$ is less than one meter, the target is regarded to be stopped at the *i*th checking points. The $Flag_{i}^{p}$ is determined as follows:

$$\begin{array}{cc} Flag_{i}^{p}=\;1 & \;(Move_{i}^{p}<1\;m)\\ Flag_{i}^{p}=\;0 & \;\;(Move_{i}^{p}>1\;m) \end{array}$$

Score of metric for naturalness of travel speed: E_v_

Given that $Flag_{i}^{v}$ is assigned zero when the target moves faster than the threshold 1.5 m/s, and one when it is below the threshold at the i^th^ frame in the trajectory’s frames.

$$E_{v}=\frac{\sum_{i=1}^{N_{traj\_frame}}Flag_{i}^{v}}{N_{traj\_frame}}\times 100$$

where N_traj_frame_ is the number of total frames of the trajectory.

Score of metric for collision with obstacles: E_o_

Given that $Flag_{i}^{o}$ is assigned one when the target pixel on the trajectory is not in the obstacle area and zero when the target pixel is in the obstacle area at the i^th^ pixels in the trajectory.

$$E_{o}=\frac{\sum_{i=1}^{N_{traj\_pixel}}Flag_{i}^{o}}{N_{traj\_pixel}}\times 100$$

where the total number of pixels in the trajectory is N_traj_pixel_.

Score of metric for position measurement output frequency: E_f_

Given that $Flag_{i}^{f}$ is assigned one when the temporal frequency is more than one Hz and zero when the frequency is less than one Hz at the *i*th frames in the submitted trajectory.

$$E_{f}=\frac{\sum_{i=1}^{N_{traj\_frame}}Flag_{i}^{f}}{N_{traj\_frame}}\times 100$$
